# Diet quality and blood pressure among adults with hypertension living in a high-altitude region of the Peruvian Andes

**DOI:** 10.1371/journal.pgph.0007396

**Published:** 2026-10-28

**Authors:** Carla Tarazona-Meza, Mika Matsuzaki, Kendra N. Williams, Laura E. Caulfield, Sabri Bromage, Lucy Cordova-Ascona, Lindsay J. Underhill, Lisa de las Fuentes, Gonzalo M. Cuentas-Canal, Zoila Vela-Clavo, Marilú Chiang, Elvin H. Geng, Victor G. Dávila-Román, Stella M. Hartinger, William Checkley

**Affiliations:** 1 Program in Human Nutrition, Department of International Health, Bloomberg School of Public Health, Johns Hopkins University, Baltimore, Maryland, United States of America; 2 Center for Global Non-Communicable Disease Research and Training, School of Medicine, Johns Hopkins University, Baltimore, Maryland, United States of America; 3 Biomedical Research Unit, A.B. PRISMA, Lima, Peru; 4 Program in Social and Behavioral Interventions, Department of International Health, Bloomberg School of Public Health, Johns Hopkins University, Baltimore, Maryland, United States of America; 5 Institute of Nutrition, Mahidol University, Nakhon Pathom, Thailand; 6 Department of Nutrition, Harvard T. H. Chan School of Public Health, Boston Massachusetts, United States of America; 7 Facultad de Salud Pública y Administración, Universidad Peruana Cayetano Heredia, Lima, Peru; 8 Centro Latino Americano de Excelencia en Cambio Climático y Salud, Universidad Peruana Cayetano Heredia, Lima, Peru; 9 Cardiovascular Division and Global Health Center, Department of Medicine, School of Medicine, Washington University, St. Louis, Missouri, United States of America; 10 Center for Biostatistics and Data Science, Institute for Informatics, Data Science and Biostatistics (I^2^BD), School of Medicine, Washington University, St. Louis, Missouri, United States of America; 11 Hospital Base III, EsSalud, Puno, Peru; 12 Infectious Diseases Division, Department of Medicine and Dissemination and Implementation Center, Institute for Public Health, Washington University in St. Louis, St. Louis, Missouri, United States of America; 13 Global Health Futures Innovation Research Network, School of Public Health, Washington University, St. Louis, Missouri, United States of America; 14 Division of Pulmonary and Critical Care Medicine, School of Medicine, Johns Hopkins University, Baltimore, Maryland, United States of America; Qatar University College of Medicine, QATAR

## Abstract

Diet quality plays an important role in the management of non-communicable diseases, including hypertension. A better understanding of dietary profiles in high-altitude populations with hypertension could help improve the effectiveness of interventions. We aimed to characterize diet quality and its association with blood pressure among adults with hypertension living at high-altitude. Cross-sectional study of baseline data from an ongoing trial in Puno, Peru. We enrolled 154 adults with hypertension participating in a community health worker-led intervention aimed at improving hypertension management in a high-altitude region of Peru. Diet was assessed by 24-hour recalls which were analyzed to calculate the Global Diet Quality Score (GDQS, range 0 – 49 with higher scores indicating better diet quality) and categorized into quartiles. We assessed blood pressure using a digital oscillometric tensiometer. We used multivariable linear regression to study the association between diet quality and blood pressure adjusted for sociodemographic characteristics, food security status, hypertension awareness and treatment, BMI, HbA1c and lifestyle habits. We further stratified the analyses by hypertension awareness and treatment. Mean ± standard deviation (SD) age of participants was 66.5 ± 10.9 years and 51.9% were females. Overall mean GDQS was 15.7 ± 3.4 points. While no statistically significant association in the overall sample in multivariable models, we found an inverse association between GDQS quartiles and systolic blood pressure (adjusted mean difference of -4.13 mmHg, 95% CI: -7.72 to -0.53 p-trend = 0.03) among participants who were aware of having hypertension and receiving treatment (n = 102). We found that study participants had generally low diet quality based on GDQS. Among those aware of and receiving treatment for hypertension, higher GDQS scores were associated with lower systolic blood pressure.

## Introduction

Hypertension is one of the most prevalent and modifiable risk factors for cardiovascular disease, including heart disease, stroke, and kidney failure, which affect billions of people globally and leads to significant morbidity and mortality [1]. Management of hypertension is crucial to prevent end organ damage and ultimately premature mortality. In low-and-middle income countries (LMICs), greater limitations on access to antihypertensive medications and timely health services than in high-income countries may lead to higher mortality from hypertension complications at younger ages [2]. Pharmacologic treatments are essential for controlling hypertension, but evidence highlights the role of diet in managing blood pressure and improving long-term cardiovascular health especially in low-resource settings where access to antihypertensive medicine and health care are limited [3–5].

Diet quality is a key determinant of overall health status. Higher diet quality is characterized by more frequent consumption of foods and drinks that provide essential nutrients and limited consumption of those that contain certain ingredients (e.g., sodium, added sugar, and saturated and *trans* fats) [6]. Diet quality can be measured using specific indicators like the Minimum Dietary Diversity for Women or the Healthy Eating Index, where a high-quality diet provides adequate amounts of nutrients, and/or limits excessive consumption of certain foods that can contribute to non-communicable diseases, respectively [6,7]. Diet quality is a crucial modifiable lifestyle factor in the management of hypertension. Indeed, a healthy diet may have similar effects as pharmacological treatment on controlling blood pressure at early disease stages [8,9].

Different dietary interventions have been evaluated to determine their effectiveness on the management of hypertension primarily in high-income country settings [10,11]. Studies have shown that adherence to the Dietary Approaches to Stop Hypertension (DASH) diet reduced systolic and diastolic blood pressure in both normotensive and hypertensive individuals, with greater effects in the latter [12,13]. The adoption of other dietary patterns such as the Mediterranean diet, characterized by a higher intake of olive oil, fish, whole grains and fresh foods, has also been reported to lead to favorable outcomes in blood pressure regulation [14,15]. Notably, dietary patterns in high-altitude settings appear to have different features than those at sea level given the unique environmental conditions, cultural traditions and lifestyle of people living at high-altitude [16,17]. The dietary pattern in Peruvian high-altitude population is characterized by high-starch and low-fat foods with low diversity of food groups [17]. However, the relative importance of dietary factors, specifically diet quality, associated with hypertension management, is largely unknown for this population.

While several metrics are available to evaluate diet quality, there remains a need to assess it in relation to both nutrient deficiency and the risk of non-communicable disease (NCD)-related health outcomes simultaneously [18]. Considering the ongoing nutrition transition, shifts in dietary patterns and physical activity in high-altitude settings from LMICs, including Peru, the novel food-based Global Diet Quality Score, previously evaluated for validity in various LMICs, offers a robust metric for evaluating diet quality in these settings [18]. The GDQS captures several relevant food groups of the DASH and Mediterranean diet, including fruits, vegetables, whole grains, legumes/nuts, and low-fat dairy, but unlike DASH, it does not directly score sodium intake or potassium-rich foods, since it is a food-group-based metric rather than a nutrient-based one.

A better understanding of context-specific evidence including specific food items, dietary profiles and food choice in high-altitude populations with a distinct food culture, could inform existing or novel interventions to optimize the control of hypertension and diabetes, either at the individual or population level. Here, we aimed to characterize diet quality, based on the Global Diet Quality Score (GDQS), and evaluate its association with blood pressure among adults with hypertension living at high-altitude.

## Methods

### Ethics statement

This study was conducted in accordance with the Declaration of Helsinki. The Johns Hopkins University School of Medicine Institutional Review Board (IRB00400041) and the Research Ethics Committee of Universidad Peruana Cayetano Heredia in Lima, Peru (212276), approved all study procedures involving participants. All participants provided written informed consent.

The parent ANDES trial is registered at ClinicalTrials.gov (NCT05524987). Its protocol was approved by the Research Ethics Committee of Universidad Peruana Cayetano Heredia (104372) and the Washington University in St. Louis Human Research Protections Office (202108158). The parent protocol was also registered with the Johns Hopkins University School of Medicine Institutional Review Board.

### Study design

This cross-sectional study was conducted in a sample of participants from the Addressing hypertension and Diabetes using community-Engaged Systems (ANDES) randomized controlled trial at baseline between February 15, and August 31, 2024 [19]. Briefly, the parent ANDES trial aims to evaluate the implementation and effectiveness of a hypertension control strategy delivered by community health workers in Puno, a southwestern Andean region of Peru located at 3,825 meters above sea level. The region is inhabited by more than 70% of Aymara and Quechua descendants. [19]. Baseline study assessments were collected as part of the ANDES trial and leveraged for this ancillary study [19].

### Study participants

We recruited 154 adult participants of the ANDES trial at baseline aged ≥18 years with hypertension defined as i) physician-prescribed use of antihypertensive medications; and/or, ii) hypertensive blood pressure measurement (systolic blood pressure [SBP] ≥ 140 mmHg and/or diastolic blood pressure [DBP] ≥ 90 mmHg) documented or measured on two or more separate occasions [19]. Participants were invited to receive additional household visits to complete the dietary intake questionnaires on a rolling basis after completing the baseline assessments.

The estimated sample size was 152 participants based on the difference detection in systolic blood pressure of 5 mmHg across diet quality levels, 95% of confidence, 80% of power and a standard deviation of 11mHg, based on previous evidence about the impact of dietary factors on blood pressure [10]. Enrollment ended when the target sample size was reached.

### Measurement of dietary intake

Dietary intake information was collected through 24-hour recalls following the multiple-pass method [20] administered by the first author and three local professional nutritionists, previously trained on standardized data collection techniques and who were familiar with local dietary patterns, recipes, and food preparation techniques.

Participants were visited at their households and guided to describe what foods and beverages were consumed both individually and in shared meals the previous day either at home or elsewhere, as well as the recipes and ingredients for mixed dishes. During the first visit, food items or meals available in the household were directly weighed using a Opalux 5000-g d-1g scale (Op-1811a-wh, Lima, Peru). Otherwise, participants were asked to identify portion sizes visually using a booklet with standardized portion sizes and to-scale pictures of common food items, plate-ware and corresponding weight in grams [21]. The 24-hour recalls were reviewed by the leading nutritionist (CTM) for consistency of the information, and the information was double entered into an in-house developed software (Microsoft Access 2023, Microsoft, Seattle, WA, USA) to estimate the daily final weight of food and beverage intake per participant.

### Diet quality

The Global Diet Quality Score (GDQS) was used to assess diet quality. This metric is based on the intake amounts from 25 food groups: 16 healthy (citrus fruits, deep orange fruits, other fruits, dark green leafy vegetables, cruciferous vegetables, deep orange vegetables, other vegetables, legumes, deep orange tubers, nuts and seeds, whole grains, liquid oils, fish and shellfish, poultry and game meat, low-fat dairy, eggs), 7 unhealthy (processed meat, refined grains and baked goods, sweets and ice cream, sugar-sweetened beverages, juice, white roots and tubers, purchased deep fried foods), and 2 unhealthy if eaten excessively (high-fat dairy, red meat) with the final score ranging from 0 to 49 points with higher GDQS total score indicating better diet quality [18].

Food and beverage weights, in the form they were consumed reported in a single 24-hour recall, were tabulated within the GDQS food groups and scored according to Bromage et al. and the INTAKE guidelines [18]. Using pre-defined food groups cutoff values of daily consumed amounts (g/day) by levels of consumption (low, middle, high or very high), healthy food groups were positively scored assigning higher points for higher consumption, whereas unhealthy and unhealthy if eaten excessively food groups were inversely scored with lower points for higher consumption. Specifically, native foods were assigned to GDQS groups following the INTAKE team standardized guideline as follows, most whole Andean grains including quinoa, kiwicha and cañihua were classified as whole grains when consumed as it, while most of Andean tubers were classified as white roots and tubers. Further details are described elsewhere [18].

The GDQS total score was then categorized into GDQS ≥ 23 (low risk), 15 – 22 (moderate risk), and <15 (high risk), following cutoff values previously associated with nutrient inadequacy and NCD-related outcomes in LMICs settings [18]. We also categorized the total GDQS into quartiles based on the distribution of scores among the trial participants, with quartile 1 representing the lowest diet quality scores and quartile 4 the highest.

### Blood pressure assessment

We asked participants to refrain from exercise, use of tobacco products, alcohol or caffeinated beverages, and cooking using biomass for 30 minutes prior to the assessment of blood pressure and confirm that they did not have a full bladder. Any heavy clothing was removed to expose the right upper arm, and an appropriately sized cuff was used. Before starting the measurement,

participants were instructed to sit on a chair in a quiet room for five minutes with legs uncrossed, back supported by a chair, and right arm supported on a table at the level of the heart (mid-sternum). We conducted three blood pressure measurements using a digital tensiometer (HEM 907XL, Omron, Kyoto, Japan) with at least 1 minute of rest between measurements [19]. The average between the second and third measurements was calculated and used for this analysis.

### Hypertension awareness and treatment

Participants with disease awareness and treatment were defined as those who answered “yes” to both of the following questions on the ANDES trial screening questionnaire: (i) “have you ever been diagnosed with high blood pressure by a physician?” and (ii) “are you currently taking medication for high blood pressure?” and without disease awareness and treatment, if any of the answers were “no” [1,19].

### Hypertension control

Hypertension control was defined as SBP/DBP < 140/90 mmHg among those participants with disease awareness and treatment [1].

### Glycemia assessment

A 5 µL finger prick blood sample was collected to evaluate Hemoglobin A1c (HbA1c) using the A1CNOW® Self check, an FDA-approved point of care device (POC-HbA1c). We used HbA1c as a measure of average glycemia over the preceding 2–3 months [19,22].

### Assessment of potential confounders

We recorded sociodemographic variables: sex, primary language, years of education, socioeconomic status, occupation and insurance type. Wealth scores were estimated from asset ownership questions using The Equity Tool [23].

We assessed food insecurity using the eight-item Food and Agriculture Organization (FAO) Food Insecurity Experience Scale (FIES) questionnaire which classifies the food insecurity status of participants based on their difficulties in accessing food due to a lack of resources over the prior 12 months [24]. Severity of food insecurity was estimated by summing the response raw scores and classifying them into food security (0 points), mild food insecurity (1 – 3 points), moderate food insecurity (4 – 6 points), and severe food insecurity (7 – 8 points) [24].

We assessed hypertension treatment adherence using the Hill-Bone Compliance Scale for adherence to antihypertensive medications and treatment advice among those participants with disease awareness and treatment [19,25]. The scale has fourteen questions about three behavioral domains related to blood pressure management: i) reduced sodium intake, ii) appointment keeping, and iii) taking of medication.

We measured height and weight three times using a stadiometer on a flat surface (SECA 213, SECA, Hamburg, Germany) and digital balance (SECA 803, SECA, Hamburg, Germany), respectively. We calculated body mass index (BMI) by dividing the final average of weight in kilograms by average of height in meters squared (kg/m^2^); and classified participants as underweight (<18.5 kg/m^2^), normal weight (18.5 – < 25 kg/m^2^), overweight (25 – < 30 kg/m^2^) and obese (≥30 kg/m^2^) according to the WHO cutoff values.

We applied the International Physical Activity Questionnaire (IPAQ) to evaluate each participant’s physical activity using the scoring system divided into work, active transportation, domestic and garden work, and leisure-time domains, and estimated their continuous score expressed as MET-min per week with higher values for more physical activity [26]. Outliers of physical activity values were defined as having a total sum across domains above 960 minutes (16 hours) following the IPAQ scoring protocol. We used the WHO ten-question Alcohol Use Disorders Identification Test (AUDIT) to determine the alcohol consumption of each participant with a higher score indicating more hazard or harmful drinking, and dependence on alcohol use [27], and the Global Adult Tobacco Survey (GATS) to quantify tobacco smoking [28].

### Statistical analysis

We conducted descriptive analyses to summarize participant characteristics by quartiles of GDQS. Continuous variables are presented as the mean ± standard deviation (SD), and categorical variables as number (percentage). We verified normal distribution of blood pressure (SBP/DBP) and diet quality using the Shapiro-Wilk test and assumptions for linearity graphically between GDQS and blood pressure. We then built multivariable linear regressions adjusted by confounders to estimate the association between blood pressure (SBP/DBP) and diet quality as a continuous score and by quartiles of the distribution. Final model selection was based on Akaike Information Criterion (AIC) goodness of fit. *A priori* confounders that we evaluated included age, sex, wealth score, years of education, occupation, food insecurity status, disease awareness and treatment, BMI, physical activity, alcohol consumption and HbA1c.

We further conducted exploratory analyses by stratifying the primary analyses across hypertension awareness and treatment status given the potential differences across these groups regarding their lifestyle modifications and medication. Additional analyses included a multivariable logistic regression to evaluate the association between diet quality and hypertension control, further adjusted for hypertension treatment adherence score. Data analyses were conducted in STATA 19 (Stata Corp., TX, USA) and graphics were created using R Studio Version 4.5.0 (2025).

## Results

### Participants characteristics

A total of 154 participants contributed data to this analysis. The mean ± SD age was 66.5 ± 10.9 years, 51.9% were female, and most participants spoke Spanish as their primary language (92.2%). Most had health insurance through former or current formal employment (62.3%), and 61% had a high school education (Table 1). Five participants had physical activity outlier values and were excluded for the primary analyses.

**Table 1:** Participant sociodemographic and lifestyle characteristics by GDQS quartiles in ANDES trial in 2024.

Participants characteristics	Total (n = 154)	Quartile 1 (n = 40) (GDQS 8.3 - 13.3)	Quartile 2 (n = 42) (GDQS 13.5 - 15.5)	Quartile 3 (n = 36) (GDQS 15.8 - 18.5)	Quartile 4 (n = 36) (GDQS 18.8 - 28.3)
**Age, years** Mean (SD)	66.5 (10.9)	67.3 (10.8)	64.0 (10.8)	70.7 (10.5)	64.4 (10.7)
**Sex** Male, % (n) Female, % (n)	48.1 (74) 51.9 (80)	55.0 (22) 45.0 (18)	42.9 (18) 57.1 (24)	47.2 (17) 52.8 (19)	47.2 (17) 52.8 (19)
**Education level** <High school, % (n) High school, % (n) <Technical or university, % (n) Technical or university, % (n)	39.6 (61) 14.3 (22) 7.8 (12) 38.3 (59)	30.0 (12) 12.5 (5) 5.0 (2) 52.5 (21)	35.7 (15) 19.1 (8) 7.1 (3) 38.1 (16)	61.1 (22) 2.8 (1) 5.6 (2) 30.6 (11)	33.3 (12) 22.2 (8) 13.9 (5) 30.6 (11)
**Health insurance** National government insurance*, % (n) Social Security system**, % (n)	37.7 (58) 62.3 (96)	30.0 (12) 70.0 (28)	33.3 (14) 66.7 (28)	50.0 (18) 50.0 (18)	38.9 (14) 61.1 (22)
**Wealth quintile** Quintile 1, % (n) Quintile 2, % (n) Quintile 3, % (n) Quintile 4, % (n) Quintile 5, % (n)	2.6 (4) 5.2 (8) 13.6 (21) 35.7 (55) 42.9 (66)	2.5 (1) 2.5 (1) 17.5 (7) 30.0 (12) 47.5 (19)	0 9.5 (4) 11.9 (5) 38.1 (16) 48.5 (17)	2.8 (1) 8.3 (3) 13.9 (5) 30.6 (11) 44.4 (16)	5.6 (2) 0 11.1 (4) 44.4 (16) 38.9 (14)
**Food insecurity** Food secure, % (n) Mild food insecure, % (n) Moderate food insecure, % (n) Severe food insecure, % (n)	48.7 (75) 24.7 (38) 16.2 (25) 10.4 (16)	52.5 (21) 22.5 (9) 12.5 (5) 12.5 (5)	38.1 (16) 28.6 (12) 23.8 (10) 9.5 (4)	58.3 (21) 16.7 (6) 13.9 (5) 11.1 (4)	47.2 (17) 30.6 (11) 13.9 (5) 8.3 (3)
**Alcohol use, points**^#^ Mean (SD)	2.96 (5.1)	4.48 (6.9)	2.64 (4.0)	2.31 (4.7)	2.31 (3.8)
Tobacco use Less than daily, % (n) Not at all, % (n)	1.9 (3) 98.1 (151)	2.5 (1) 97.5 (39)	0 (0) 100.0 (42)	2.8 (1) 97.2 (35)	2.8 (1) 97.2 (35)
	**(n = 149)**	**(n = 39)**	**(n = 40)**	**(n = 35)**	**(n = 35)**
**Physical Activity, MET-min/week**^‡^ Median (IQR)	1726.5 (602 - 4473)	732 (345 – 4179)	2196.5 (933 – 4407)	3960 (866 – 4833)	1726.5 (755 – 4377)

*Public insurance, Comprehensive Health Insurance Scheme for individuals in poverty (SIS); **Social Security system - employer provided (EsSalud)

# Higher points to alcohol use indicate a higher use

‡ A higher the MET-min/week, indicating more physical activity

Abbreviations: GDQS, Global Diet Quality Score; SD, Standard Deviation

Nearly all participants were either overweight (40.9%) or obese (46.1%). Mean SBP and DBP were 133.8 ± 18.2 mmHg and 76.9 ± 12.3 mmHg, respectively. Over half (66.2%) of participants were aware of having hypertension and receiving treatment; participants with hypertension awareness and treatment were older than those who were not (p = 0.03). Out of 102 participants with disease awareness and treatment, 67.7% had controlled hypertension and mean treatment adherence score was 49 ± 3.4 out of 56 points. Mean ±SD HbA1c was 6.1% ± 1.0 (Table 2). Median (Interquartile range, IQR) daily energy intake in participants was 1,305 (IQR: 854 – 1,689) kcal/day (Table A in S1 Appendix).

**Table 2:** Participant clinical characteristics by GDQS quartiles in ANDES trial in 2024.

Clinical characteristics	Total (n = 154)	Quartile 1 (n = 40) (GDQS 8.3 - 13.3)	Quartile 2 (n = 42) (GDQS 13.5 - 15.5)	Quartile 3 (n = 36) (GDQS 15.8 - 18.5)	Quartile 4 (n = 36) (GDQS 18.8 - 28.3)
**BMI (kg/m^2^)** Normal, % (n) Overweight, % (n) Obesity, % (n)	12.9 (20) 40.9 (63) 46.1 (71)	10.0 (4) 40.0 (16) 50.0 (20)	9.5 (4) 42.9 (18) 47.6 (20)	19.4 (7) 44.4 (16) 36.1 (13)	13.9 (5) 36.1 (13) 50.0 (18)
**Blood pressure (mmHg)** SBP, Mean (SD) DBP, Mean (SD)	133.84 (18.2) 76.89 (12.3)	138.84 (17.8) 80.73 (11.1)	130.31 (18.6) 75.36 (12.9)	131.29 (19.4) 73.99 (11.8)	134.94 (16.4) 77.36 (12.7)
**Hypertension awareness and treatment** No, % (n) Yes, % (n)	33.8 (52) 66.2 (102)	37.5 (15) 62.5 (25)	26.2 (11) 73.8 (31)	30.6 (11) 69.4 (25)	41.7 (15) 58.3 (21)
	**(n = 102)**	**(n = 25)**	**(n = 31)**	**(n = 25)**	**(n = 21)**
Hypertension control* Uncontrolled, % (n) Controlled, % (n)	32.4 (33) 67.6 (69)	44.0 (11) 56.0 (14)	32.3 (10) 67.7 (21)	20.0 (5) 80.0 (20)	33.3 (7) 66.7 (14)
**Hill-Bone Scale, points** Mean (SD)	49.0 (3.4)	49.9 (2.5)	48.3 (3.6)	48.0 (3.9)	50.1 (2.6)
	**(n = 147)**	**(n = 37)**	**(n = 40)**	**(n = 35)**	**(n = 35)**
**Glycemia HbA1c %** Mean (SD)	6.08 (1.0)	6.11 (0.9)	6.15 (1.3)	5.96 (0.7)	6.08 (1.1)

* Hypertension control using the SBP/DBP < 140/90 mmHg threshold among those with prior hypertension diagnosis and treatment

Abbreviations: GDQS, Global Diet Quality Score; BMI, body mass index; SBP, systolic blood pressure; DBP, diastolic blood pressure; HbA1c, Hemoglobin A1c test; SD, Standard Deviation

### Diet quality

We summarized the intake of each GDQS food group in Table 3. The overall mean GDQS was 15.7 ± 3.4 out of 49 points with no differences by sex (p = 0.41) or age (p = 0.47). Only two participants (1.3%) had low risk (>23 points) of malnutrition, while 55.8% and 42.9% had moderate (15–23 points) and high risk (<15 points) of malnutrition, respectively.

**Table 3:** GDQS food group mean daily consumption of participants in ANDES trial 2024.

GDQS Food groups	Number of respondents >0 g	Amounts (g/day) Mean (SD)
**Healthy Food groups (GDQS+)**		
Citrus fruits	67	118.6 (120.2)
Deep orange fruits	1	432.6
Other fruits	64	216.9 (197.1)
Dark green leafy vegetables	50	25.9 (59.9)
Cruciferous vegetables	21	15.2 (19.4)
Deep orange vegetables	117	51.6 (59.4)
Other vegetables	140	95.3 (103.8)
Legumes	34	38.5 (43.9)
Deep orange tubers	3	302.3
Nuts and seeds	13	2.5 (6.7)
Whole grains	91	265.4 (282.7)
Liquid oils	79	17.9 (43.8)
Fish and shellfish	21	86.7 (74.4)
Poultry and game meat	85	97.7 (88.9)
Low-fat dairy	2	31.9
Eggs	50	58.3 (43.3)
**Unhealthy in excessive amounts food groups (GDQS-)**		
High-fat dairy	68	126.3 (180.8)
Red meat	63	68.1 (70.0)
**Unhealthy food groups (GDQS-)**		
Processed meat	3	6.8
Refined grains and baked goods	137	198.4 (172.9)
Sweets and ice cream	129	36.7 (51.7)
Sugar-sweetened beverages	32	346.4 (336.8)
Juice	24	408.5 (179.3)
White roots and tubers	135	262.4 (275.4)
Purchased deep fried foods	14	148.92 (91.9)

Means and Standard Deviation (SD) calculated among consumers only (n > 0g day). SD not shown for food items consumed by less than 5 participants.

Abbreviations: GDQS, Global Diet Quality Score; SBP, systolic blood pressure; DBP, diastolic blood pressure

The mean score for healthy food groups was 5.9 ± 2.8 out of 32 points, and for unhealthy food groups was 9.8 ± 1.8 out of 17 points. Low scores for healthy food groups were due to low consumption of deep orange fruits, cruciferous vegetables or deep orange tubers, and low-fat dairy foods. Furthermore, high scores for unhealthy food groups were due to low consumption of processed meat, sugar-sweetened beverages, and purchased deep-fried foods. Importantly, white roots and tubers, refined grains and baked goods had a high and middle consumption in 80% of participants.

More than 50% of participants had a high intake level for each food group of whole grains, refined grains and baked goods, or white roots and tubers (Fig 1 and Table B in S1 Appendix).

**Fig 1:**
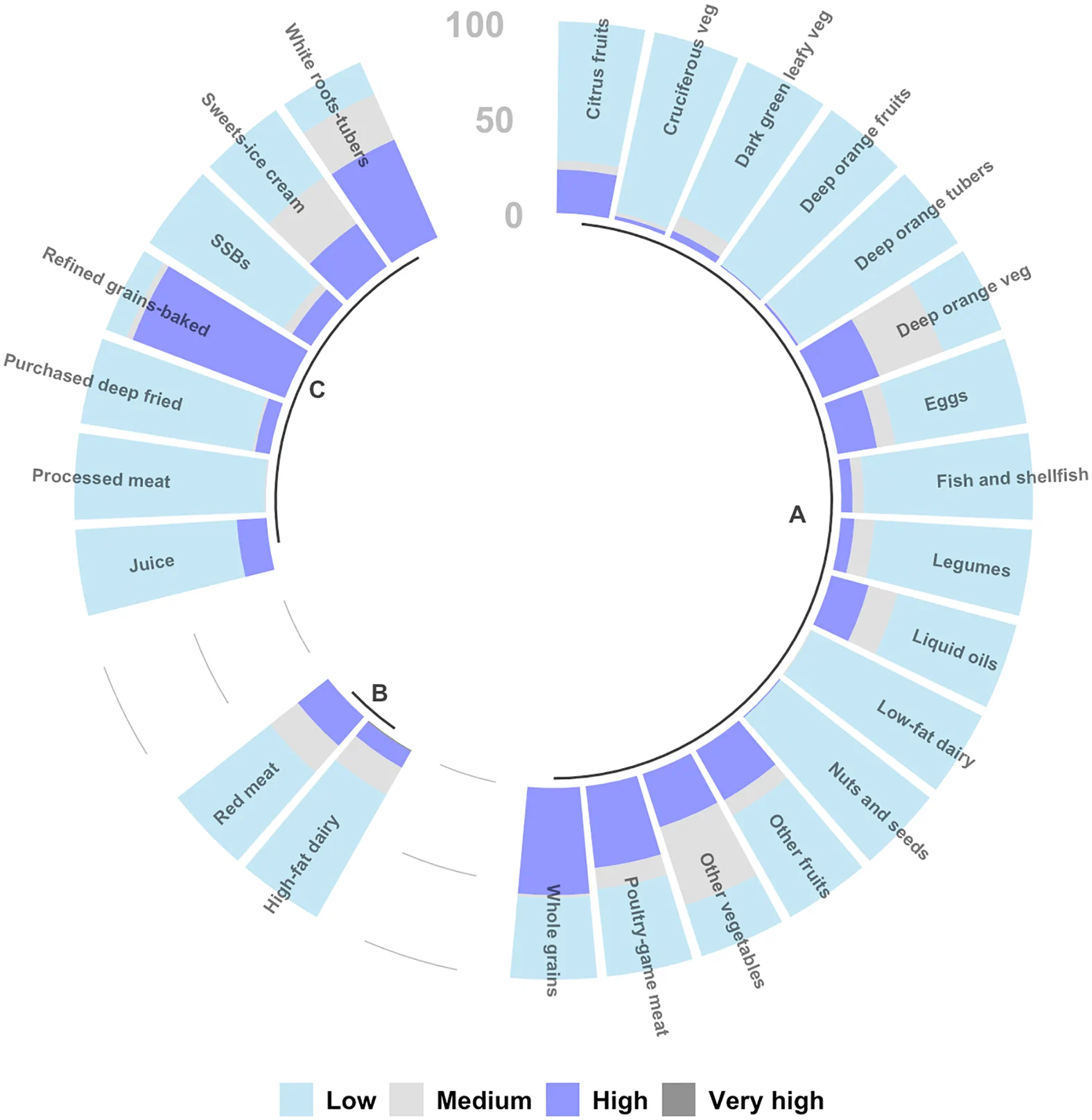
Proportion of participants by GDQS levels of intake of all food groups in ANDES trial in 2024. GDQS food groups: A. GDQS+ (healthy); B. GDQS- (unhealthy if eaten excessively); C. GDQS- (unhealthy). Proportion of participants (0 – 100%) with low intake (sky-blue), moderate or medium intake (gray), high intake (purple), and very high intake (black) of 25 food groups according to GDQS cutoff values.

We further display the mean scores of GDQS, GDQS+ healthy foods consumption and GDQS- unhealthy foods consumption by risk of malnutrition categories and GDQS quartiles in Fig 2 and Fig 3.

**Fig 2:**
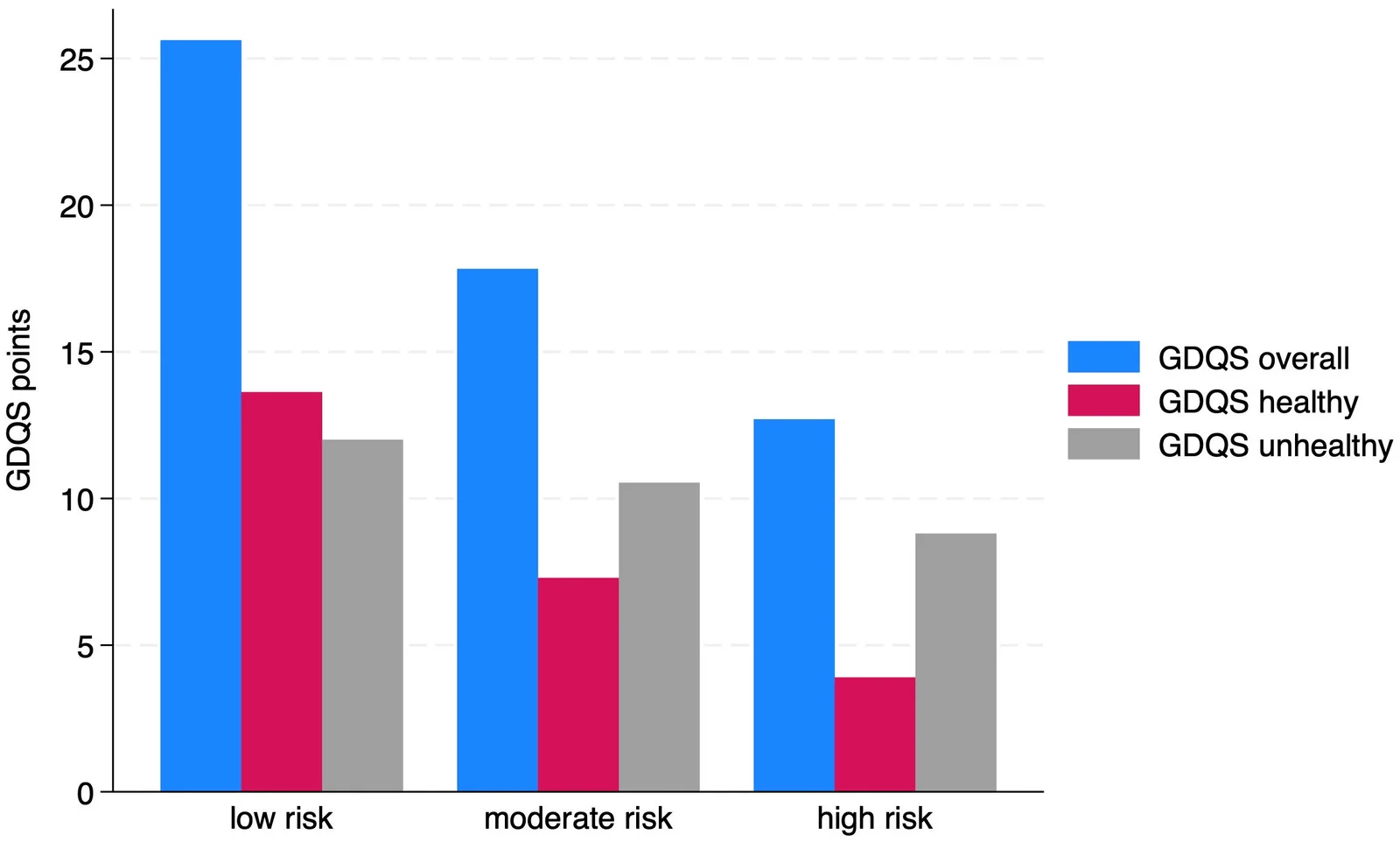
Global Diet Quality Scores means across categories of risk to nutrient deficiency and NCD-related outcomes. *GDQS categories of risk to nutrient deficiency and NCD-related outcomes**:** Low (>23 points), moderate (15 – 23 points), and high (<15 points).

**Fig 3:**
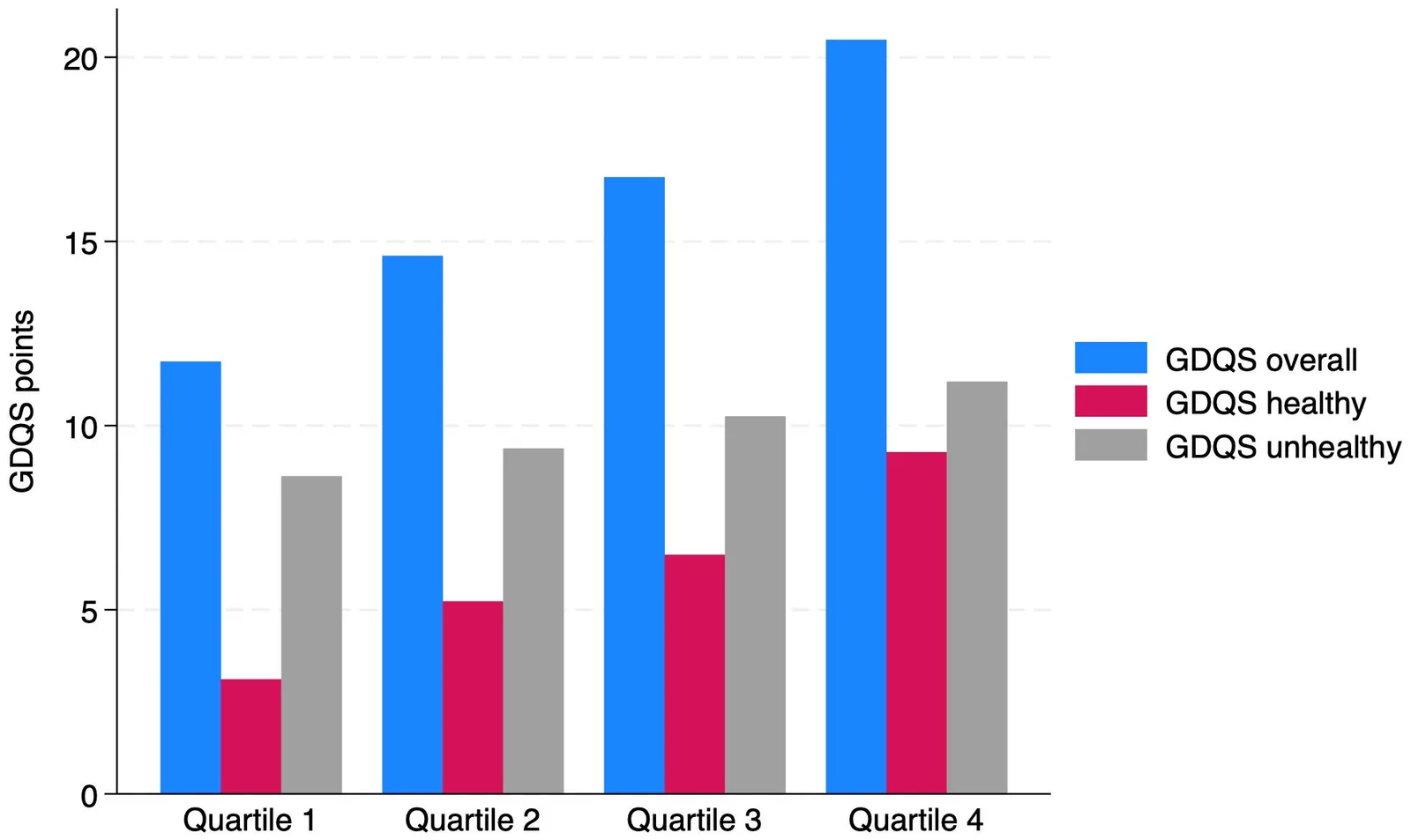
Global Diet Quality Scores means across GDQS quartiles. *GDQS quartiles: Quartile 1 (8.3, 13.3 points); Quartile 2 (13.5, 15.5 points); Quartile 3 (15.8, 18.5 points); Quartile 4 (18.8, 28.3 points).

### Association between diet quality and blood pressure

We did not find associations between GDQS continuous score and SBP (adjusted mean difference by 1 point of GDQS of -0.68 mmHg, 95% CI: -1.59, 0.25; p = 0.15) or DBP (adjusted mean difference by 1 point of GDQS of -0.42 mmHg, 95% CI: -1.03, 0.17; p = 0.17).

Similarly, Table 4 shows that GDQS quartiles were not associated with SBP (adjusted mean difference by GDQS quartile of -2.07 mmHg, 95% CI: -4.87, 0.74; p-trend = 0.15) or DBP (adjusted mean difference by GDQS quartile of -1.48, 95% CI: -3.31, 0.36; p-trend = 0.11) in all the study participants.

**Table 4:** Association between diet quality quartiles and blood pressure in ANDES trial 2024.

	Quartile 1	Quartile 2	Quartile 3	Quartile 4	p-trend
***Systolic Blood Pressure (mmHg)***					
**Unadjusted** ^‡^	Reference	**-8.5 (-16.4, -0.6)**	-7.5 (-15.8, 0.7)	-3.9 (-12.1, 4.3)	0.40
Adjusted* ^‡^	Reference	-8.8 (-17.6, 0.1)	**-13.6 (-22.6, -4.5)**	-5.9 (-14.7, 2.9)	0.15
***Diastolic Blood Pressure (mmHg)***					
**Unadjusted** ^‡^	Reference	**-5.4 (-10.7, -0.1)**	**-6.7 (-12.2, -1.2)**	-3.4 (-8.9, 2.1)	0.19
Adjusted* ^‡^	Reference	**-6.9 (-12.7, -1.1)**	**-7.7 (-13.7, -1.8)**	-5.1 (-10.8, 0.6)	0.11

Diet quality scores: Quartile 1 (8.3, 13.3 points); Quartile 2 (13.5, 15.5 points); Quartile 3 (15.8, 18.5 points); Quartile 4 (18.8, 28.3 points)

*Adjusted models for age, sex, wealth score, food security status, years of education, occupation, hypertension awareness and treatment, BMI, physical activity, alcohol intake and glycemia HbA1c

‡ β-coefficient (95% Confidence Interval) of linear regressions

### Exploratory analyses by hypertension awareness and treatment

We did not identify a statistically significant interaction between GDQS continuous and hypertension awareness and treatment for SBP (p = 0.40) or DBP (p = 0.74).

Among those without hypertension awareness and treatment (n = 48), there was no association between GDQS continuous score and either SBP (adjusted mean difference by 1 point of GDQS of 0.24 mmHg, 95% CI: -1.46, 1.93; p = 0.78) or DBP (adjusted mean difference by 1 point of GDQS of 0.25 mmHg, 95% CI: -0.86, 1.38; p = 0.65). Nevertheless, among those with hypertension awareness and treatment (n = 91), we found a negative association between a higher continuous GDQS score (better diet quality) and lower SBP (adjusted mean difference by 1 point of GDQS -1.28 mmHg, 95% CI: -2.55, -0.001; p = 0.04) (Table C in S1 Appendix).

We have also identified a negative association between higher GDQS quartiles and lower SBP (adjusted mean difference by GDQS quartile of -4.13 mmHg, 95% CI: -7.72, -0.53; p-trend = 0.03) and borderline significant for DBP (adjusted mean difference by GDQS quartile of -2.27 mmHg, 95% CI: -4.53, -0.01; p-trend = 0.05) among those with hypertension awareness and treatment. Results across quartiles by each stratum are shown in Table 5.

**Table 5:** Association between diet quality quartiles and blood pressure by hypertension awareness and treatment in ANDES trial 2024.

	Quartile 1	Quartile 2	Quartile 3	Quartile 4	p-trend
Not aware of and treated for hypertension *					
SBP (mmHg)^‡^	Reference	-9.5 (-28.3, 9.3)	-8.9 (-30.1, 12.3)	1.5 (-16.3, 19.3)	0.56
DBP (mmHg) ^‡^	Reference	-9.4 (-17.5, 2.9)	-3.7 (-17.5, 10.2)	0.1 (-11.5, 11.8)	0.52
Aware of and treated for hypertension *					
SBP (mmHg) ^‡^	Reference	**-11.0 (-22.1, -0.1)**	**-18.2 (-29.6, -6.8)**	-11.3 (-22.7, 0.2)	**0.03**
DBP (mmHg) ^‡^	Reference	-5.6 (-12.7, 1.4)	**-8.4 (-15.7, -1.1)**	-6.6 (-13.9, 0.7)	**0.05**

Diet quality scores: Quartile 1 (8.3, 13.3 points); Quartile 2 (13.5, 15.5 points); Quartile 3 (15.8, 18.5 points); Quartile 4 (18.8, 28.3 points)

*Adjusted models for age, sex, wealth score, food security status, years of education, occupation, BMI, physical activity, alcohol intake and glycemia HbA1c and “n” individuals with complete data on covariables

‡β-coefficient (95% Confidence Interval) of linear regressions

Abbreviations: GDQS, Global Diet Quality Score; SBP, systolic blood pressure; DBP, diastolic blood pressur

### Association between diet quality and hypertension control

Among participants with hypertension awareness and treatment (n = 102), there was no clear evidence of a difference between GDQS continuous score and odds of having controlled hypertension (adjusted OR of 1.12, 95% CI: 0.95, 1.33; p = 0.19) in adjusted analysis.

## Discussion

To our knowledge, this is the first study in Peru to assess diet quality using the Global Diet Quality Score. Overall, diet quality among people with hypertension was poor. Fewer than 15% consumed deep orange fruits, cruciferous vegetables, or deep orange tubers, while about 20% consumed sugar-sweetened beverages or juices. However, we also identified low intake of unhealthy food groups with fewer than 2% of participants consuming processed meat. Although no statistically significant association was found between diet quality and blood pressure, higher quartiles of diet quality score were linked to lower systolic and diastolic blood pressure compared to the lowest quartile particularly among individuals who were aware of having hypertension and receiving treatment.

The Global Diet Quality Score has been used in other LMICs, with similar mean scores reported in Brazil (14.5 points), Mexico (16.4 points), and Vietnam (17.9 points) [29–31]. In contrast, a study in India found a higher mean score of 23 among nonpregnant women of reproductive age [32]. While diet quality is often higher in women than men, as seen in Brazil and Australia [29,33], our study found no clear sex-based differences in GDQS.

Overall, participants in our study were primarily older adults and either overweight or obese, which may partially explain their low diet quality scores mostly classified as moderate and high risk to malnutrition. Studies in Indonesia and the USA using different metrics for diet quality also reported poor diet quality scores among elderly populations [34,35]. Using the American Heart Association score and the Healthy Eating Index-2015, they reported low intake of fruits, vegetables and whole grains among older adults [34,35]. Further, higher socioeconomic status has been associated with better diet quality and diversity [36], which may provide another potential explanation for our findings, given that less than half of our participants were food secure. Given that most participants were at moderate to high risk of malnutrition, further attention to potential underlying factors, such as poor appetite, chronic disease burden, or socioeconomic constraints common among older adults with hypertension, is needed. This finding underscores the importance of integrated nutritional screening in this population.

Our findings align with prior research in high-altitude settings that reported low dietary diversity among adults living in rural areas in Peru [37,38], and a dietary pattern characterized by limited consumption of ultra-processed foods [17].

A key feature of this Andean dietary pattern is high intake of potatoes, primarily boiled, achieving up to 1 kg/person/day seasonally [39]. Therefore, potatoes represent the main staple source of nutrients and minerals including potassium, zinc and protein, contributing significantly to total energy and zinc, especially for women and children [39]. Despite representing an important source of nutrients in this population, the GDQS classifies all potatoes, regardless of their cooking methods, as unhealthy foods, resulting in lower diet quality scores [18]. Notably, cooking methods may influence the potential effect of potatoes intake on cardiovascular health. A cohort study in the US reported that more than four servings a week of French fries, fried potatoes consumed with salt, was associated with a 17% higher risk of hypertension in healthy adults [40]. Another study of Spanish adults at high-risk of cardiovascular disease, including hypertension and T2DM, found that higher consumption of homemade fries, typically prepared with salt added, was associated with elevated SBP among those not receiving hypertension treatment at the baseline [41]. Consequently, potato consumption patterns in our study population may not meaningfully contribute to certain diet quality metrics due to limited variation on intake and primarily non-fried cooking method across the adult population, introducing noise that hampers differentiation by these metrics. Nonetheless, the influence of potato consumption on blood pressure warrants further investigation, both in terms of its direct effects when consumed with added salt, and indirectly, via weight gain.

Even though better diet quality, comprehensively assessed through different metrics, has been associated with lower hypertension risk and lower blood pressure in the general population [42,43], fewer studies examine the role of diet quality on hypertension management among individuals who already have hypertension in LMICs. Previous evidence reported that older populations with hypertension among other metabolic conditions, have been found to respond more positively to improvements in diet quality than younger and healthier populations [44,45].

Our study did not identify an association between GDQS and blood pressure across all participants. This null result may be due to limited variation in diet quality, as the uniformly low GDQS scores likely reduced our ability to detect meaningful differences. It is also possible that the GDQS itself had limited ability to capture meaningful variation in diet quality and, by extension, its association with SBP. Our results align with findings from rural Ethiopia, a context like ours, including adults living at high or very high altitudes, where diet quality measures also showed limited differentiation [46].

However, in exploratory analyses, we identified a lower systolic blood pressure at higher scores and quartiles of GDQS among those with hypertension awareness and treatment. One possible explanation for our findings may be the synergistic effect between a better diet quality and medication treatment on the management of blood pressure considering the earlier access to hypertension diagnosis, lifestyle recommendations and antihypertensive medication prescription in this group. Evidence from a randomized controlled trial identified a greater effect of a plant-based dietary pattern on systolic blood pressure among those using antihypertensive drugs in comparison to those not using antihypertensive medication, but no clear differences on diastolic blood pressure [47]. Another observational study in Korea found a positive association between higher diet quality scores and blood pressure control in men previously diagnosed with hypertension by a physician [48].

Another possible explanation for our findings is the potential behavioral confounding by self-management and self-motivation to schedule and attend to healthcare appointments, better adherence to medication and recommendations about lifestyle modifications given the potential co-morbidity or disease severity at older ages in this subgroup. Therefore, long-term adherence to medical recommendations along with dietary changes towards a healthier diet may have led to an inverse association with blood pressure only among those who were disease aware and treated, while not attributing solely this association to diet.

Our findings contribute valuable information to the field of hypertension management in LMICs. Given the low levels of hypertension awareness, treatment, and control in Puno [49], is crucial to implement interventions that promote early diagnosis and support nutritional changes towards healthier dietary patterns, which may help prevent complications and reduce premature mortality. Along with early disease diagnosis, current recommendations on lifestyle modifications, especially on diet, oriented to patients diagnosed with hypertension, should be reinforced with other strategies given the low diet quality identified in our study. In a context where the primary nutritional advice is provided by the health care professionals at the hospital level without continuous monitoring at the household level, nutrition education messages delivered by community health workers may help to reach more patients closely and expand this strategy more frequent. These intervention models have important beneficial effects on blood pressure management [50] and represent the primary intervention of the ANDES trial following the WHO HEARTS technical package [19] in the region. Our findings reinforce the need to integrate nutrition education messages into community-based hypertension management programs aligned with the WHO HEARTS framework. Future culturally tailored nutrition interventions should focus on improving access to healthier diets while promoting dietary diversity among the locally available foods and healthy cooking methods. Along with nutrition education, these interventions may include those focused on strengthening the local food systems and improving food environments to ensure access to healthy foods and nutritious diets to low-resource households.

This study has many strengths. To our knowledge, this is the first study in Peru to assess the association between diet quality, comprehensively assessed using a standardized method, and blood pressure among adults with hypertension living at high altitude. Previous studies have characterized local dietary patterns across the general population and have not evaluated the role of diet quality in NCDs management. In addition, we also characterized the mean and level of intake across specific Global Diet Quality Score food groups and the proportion of participants consuming each of them, providing a comprehensive understanding of the consumption patterns and the main drivers behind the low diet quality in this population. We captured dietary intakes using 24-hour recalls which provided more detailed information about food intake weights. Further, blood pressure as well as other covariates were assessed directly by trained field workers using standardized and calibrated equipment following international guidelines to decrease any potential measurement error. Our study also has some potential limitations. First, the dietary intake was self-reported, which is subject to biases due to social desirability or problems with recall (e.g., misreporting food intake or portion sizes), especially among those with higher BMI. Second, the cross-sectional study design limits the understanding of a true association between diet quality and blood pressure given that both variables were assessed at the same time, as well as, in addition to a limited GDQS variability, it may have potential upward-biased the slope and the effect size should be interpreted cautiously. We have not conducted further adjustment for multiple testing and results should be interpreted cautiously. Blood pressure usually responds to recent changes in diet and, considering the daily variation in dietary intake assessments, it may influence the effect size of a true association. Furthermore, the small sample size and recruitment method may limit the generalizability of our results to the target population, which may differ on key behaviors including a more health-engagement and Global Diet Quality Score among other groups with different characteristics like younger adults or with normal BMI. Finally, our study does not objectively assess dietary sodium which represent an important constraint when interpreting these results. Nevertheless, our study provides relevant information about the diet quality in elderly people with hypertension living at high-altitude.

## Conclusions

Diet quality was generally low in this group of participants, adults with hypertension living in a high-altitude region of Peru. The low Global Diet Quality Score was primarily driven by a low intake of healthy food groups. These findings are based on the distribution of Global Diet Quality Scores among our study participants. Larger longitudinal studies are needed to better understand how diet quality influences cardiometabolic outcomes to inform locally targeted, culturally tailored nutrition interventions aimed at improving access to healthier diets and ultimately enhancing disease management in Andean populations.

## Supporting information

S1 Appendix Table A. Daily energy intake by participants clinical characteristics in ANDES trial in 2024 (n = 154). *Hypertension control using the SBP/DBP < 140/90 mmHg threshold among those with prior hypertension diagnosis and treatment. Abbreviations: GDQS, Global Diet Quality Score; BMI, body mass index; SBP, systolic. Table B. Proportion of participants by GDQS levels of intake of all food groups in ANDES trial in 2024. Abbreviations: GDQS, Global Diet Quality Score. Table C. Association between continuous GDQS and blood pressure by hypertension awareness and treatment in ANDES trial 2024. *Adjusted models for age, sex, wealth score, food security status, years of education, occupation, BMI, physical activity, alcohol intake and glycemia HbA1c and “n” individuals with complete data on covariables. ^‡^β-coefficient (95% Confidence Interval) of linear regressions. Abbreviations: GDQS, Global Diet Quality Score; SBP, systolic blood pressure; DBP, diastolic blood pressure.
(DOCX)

## References

[1] NCD Risk Factor Collaboration (NCD-RisC). Worldwide trends in hypertension prevalence and progress in treatment and control from 1990 to 2019: a pooled analysis of 1201 population-representative studies with 104 million participants. Lancet. 2021;398(10304):957–80. doi: 10.1016/S0140-6736(21)01330-1 34450083 PMC8446938

[2] Miranda JJ, Barrientos-Gutiérrez T, Corvalan C, Hyder AA, Lazo-Porras M, Oni T, et al. Understanding the rise of cardiometabolic diseases in low- and middle-income countries. Nat Med. 2019;25(11):1667–79. doi: 10.1038/s41591-019-0644-7 31700182

[3] Mattei da Silva ÂT, de Fátima Mantovani M, Castanho Moreira R, Perez Arthur J, Molina de Souza R. Nursing case management for people with hypertension in primary health care: A randomized controlled trial. Res Nurs Health. 2020;43(1):68–78. doi: 10.1002/nur.21994 31710134

[4] Jafar TH, Gandhi M, de Silva HA, Jehan I, Naheed A, Finkelstein EA, et al. A community-based intervention for managing hypertension in rural South Asia. N Engl J Med. 2020;382(8):717–26. doi: 10.1056/NEJMoa1911965 32074419

[5] Gamage DG, Riddell MA, Joshi R, Thankappan KR, Chow CK, Oldenburg B, et al. Effectiveness of a scalable group-based education and monitoring program, delivered by health workers, to improve control of hypertension in rural India: A cluster randomised controlled trial. PLoS Med. 2020;17(1):e1002997. doi: 10.1371/journal.pmed.1002997 31895945 PMC6939905

[6] Hiza HAB, Koegel KL, Pannucci TE. Diet quality: The key to healthy eating. J Acad Nutr Diet. 2018;118(9):1583–5. doi: 10.1016/j.jand.2018.07.002 30146069

[7] FAO. Minimum dietary diversity for women. Rome: FAO; 2021. doi: 10.4060/cb3434en

[8] Appel LJ. The effects of dietary factors on blood pressure. Cardiol Clin. 2017;35(2):197–212. 10.1016/j.ccl.2016.12.002 28411894

[9] Forouhi NG, Misra A, Mohan V. Dietary and nutritional approaches for prevention and management of type 2 diabetes. BMJ. 2018;2018:k2234. 10.1136/bmj.k2234 PMC5998736 29898883

[10] Gay HC, Rao SG, Vaccarino V. Effects of different dietary interventions on blood pressure. Hypertension. 2016;67(4):733–9. 10.1161/HYPERTENSIONAHA.115.06853 26902492

[11] Aljuraiban GS, Gibson R, Chan DS, Van Horn L, Chan Q. The role of diet in the prevention of hypertension and management of blood pressure: An umbrella review of meta-analyses of interventional and observational studies. Adv Nutr. 2024;15(1):100123. doi: 10.1016/j.advnut.2023.09.011 37783307 PMC10831905

[12] Filippou CD, Tsioufis CP, Thomopoulos CG, Mihas CC, Dimitriadis KS, Sotiropoulou LI, et al. Dietary Approaches to Stop Hypertension (DASH) diet and blood pressure reduction in adults with and without hypertension: A systematic review and meta-analysis of randomized controlled trials. Adv Nutr. 2020;11(5):1150–60. doi: 10.1093/advances/nmaa041 32330233 PMC7490167

[13] Juraschek SP, Miller ER, Weaver CM. Effects of sodium reduction and the DASH diet in relation to baseline blood pressure. J Am Coll Cardiol. 2017;70(23). 10.1016/j.jacc.2017.10.011 PMC5742671 29141784

[14] León-Muñoz LM, Guallar-Castillón P, Graciani A, López-García E, Mesas AE, Taboada JM, et al. Dietary habits of the hypertensive population of Spain: Accordance with the DASH diet and the Mediterranean diet. J Hypertens. 2012;30(7):1373–82. doi: 10.1097/HJH.0b013e328353b1c1 22525205

[15] De Pergola G, D’Alessandro A. Influence of mediterranean diet on blood pressure. Nutrients. 2018;10(11):1700. doi: 10.3390/nu10111700 PMC6266047 30405063

[16] Xiao Z, Sun X, Zhaxi D, Zhang F, Ji Y, Cheng T, et al. Distinct nutrient intake style in inhabitants of ultra-high-altitude areas in north of Tibet, China: A cross-sectional study based on newly developed tibetan food frequency questionnaires. Front Nutr. 2021;8:743896. doi: 10.3389/fnut.2021.743896 35004798 PMC8733569

[17] Alae-Carew C, Scheelbeek P, Carrillo-Larco RM, Bernabé-Ortiz A, Checkley W, Miranda JJ. Analysis of dietary patterns and cross-sectional and longitudinal associations with hypertension, high BMI and type 2 diabetes in Peru. Public Health Nutr. 2020;23(6):1009–19. doi: 10.1017/S1368980019002313 31456536 PMC7613217

[18] Bromage S, Batis C, Bhupathiraju SN. Development and validation of a novel food-based global diet quality score (GDQS). J Nutr. 2021;151(Suppl 2):75S-92S. 10.1093/jn/nxab244 PMC8542096 34689200

[19] Underhill LJ, Williams KN, Cordova-Ascona L. Addressing hypertension and diabetes through community-engaged systems (ANDES) in Puno, Peru: Rationale and study protocol for a hybrid type 2 effectiveness and implementation randomized controlled trial. Trials. 2024;25(1):747. 10.1186/s13063-024-08586-9 PMC11549830 39516847

[20] Moshfegh AJ, Rhodes DG, Baer DJ. The US department of agriculture automated multiple-pass method reduces bias in the collection of energy intakes. Am J Clin Nutr. 2008;88(2):324–32. 10.1093/ajcn/88.2.324 18689367

[21] Laminario medidas caseras - PRISMA | PDF. 2023. Accessed 2026 June 19. https://es.scribd.com/document/412064981/Laminario-Medidas-Caseras-PRISMA

[22] ADA. Understanding A1C Test. 2025. Accessed 2026 June 19. https://diabetes.org/about-diabetes/a1c

[23] Equity Tool. The EquityTool. Home. Accessed June 19, 2026. https://www.equitytool.org/

[24] Food and Agriculture Organization of the United Nations. Food Insecurity Experience Scale. Voices of the Hungry. 2022. Accessed 2026 June 19. https://www.fao.org/in-action/voices-of-the-hungry/fies/en/

[25] Kim MT, Hill MN, Bone LR, Levine DM. Development and testing of the hill-bone compliance to high blood pressure therapy scale. Prog Cardiovasc Nurs. 2000;15(3):90–6. doi: 10.1111/j.1751-7117.2000.tb00211.x 10951950

[26] Craig CL, Marshall AL, Sjöström M, Bauman AE, Booth ML, Ainsworth BE, et al. International physical activity questionnaire: 12-country reliability and validity. Med Sci Sports Exerc. 2003;35(8):1381–95. doi: 10.1249/01.MSS.0000078924.61453.FB 12900694

[27] World Health Organization, Babor TF, Higgins-Biddle JC, Saunders JB, Monteiro MG. AUDIT: the alcohol use disorders identification test: Guidelines for use in primary health care. 2nd ed. World Health Organization; 2001.

[28] Global Adult Tobacco Survey Collaborative Group. Global Adult Tobacco Survey (GATS): Fact Sheet Templates. Atlanta, GA: Centers for Disease Control and Prevention; 2020. https://cdn.who.int/media/docs/default-source/ncds/ncd-surveillance/gats/18_gats_analysispackage_final_23nov2020.pdf?sfvrsn=67e2065f_4

[29] Norde MM, Bromage S, Marchioni DML. The global diet quality score as an indicator of adequate nutrient intake and dietary quality – a nation-wide representative study. Nutr J. 2024;23(1):42. 10.1186/s12937-024-00949-x PMC11022474 38627669

[30] Castellanos-Gutiérrez A, Rodríguez-Ramírez S, Bromage S. Performance of the global diet quality score with nutrition and health outcomes in Mexico with 24-h recall and FFQ data. J Nutr. 2021;151(Supplement_2):143S-151S. 10.1093/jn/nxab202 PMC8542100 34689195

[31] Nguyen PH, Tran LM, Hoang NT, Deitchler M, Moursi M, Bergeron G. The Global diet quality score is associated with nutrient adequacy and depression among Vietnamese youths. Ann N Y Acad Sci. 2023;1528(1):48–57. doi: 10.1111/nyas.15053 37566812

[32] Matsuzaki M, Birk N, Bromage S. Validation of global diet quality score among nonpregnant women of reproductive age in India: Findings from the Andhra Pradesh Children and Parents Study (APCAPS) and the Indian Migration Study (IMS). J Nutr. 2021;151(12 Suppl 2):101S-109S. 10.1093/jn/nxab217 PMC8564710 34689191

[33] Fenton S, Ashton LM, Lee DCW, Collins CE. Gender differences in diet quality and the association between diet quality and BMI: An analysis in young Australian adults who completed the Healthy Eating Quiz. J Hum Nutr Diet. 2024;37(4):943–51. doi: 10.1111/jhn.13309 38664922

[34] Surya R, Surya E, Nurkolis F, Santos RS, Suryani Y. Better diet quality is associated with healthier aging among urban elderly. Nutrition and Healthy Aging. 2023;8(1):123–30. doi: 10.3233/nha-220178

[35] Long T, Zhang K, Chen Y. Trends in diet quality among older US adults from 2001 to 2018. JAMA Netw Open. 2022;5(3):e221880. doi: 10.1001/jamanetworkopen.2022.18880 PMC8917422 35275167

[36] Mayén A-L, Marques-Vidal P, Paccaud F, Bovet P, Stringhini S. Socioeconomic determinants of dietary patterns in low- and middle-income countries: A systematic review. Am J Clin Nutr. 2014;100(6):1520–31. doi: 10.3945/ajcn.114.089029 25411287

[37] McCloskey ML, Tarazona-Meza CE, Jones-Smith JC, Miele CH, Gilman RH, Bernabe-Ortiz A, et al. Disparities in dietary intake and physical activity patterns across the urbanization divide in the Peruvian Andes. Int J Behav Nutr Phys Act. 2017;14(1):90. doi: 10.1186/s12966-017-0545-4 28693514 PMC5504673

[38] Tarazona-Meza C, Williams KN, Malpartida G, Kephart JL, Fandiño-Del-Río M, Simkovich S, et al. Effects of a household air pollution intervention using liquefied petroleum gas stoves, continuous fuel distribution and behavioural messaging on dietary and sodium intake of adult women in Puno, Peru: A randomised controlled trial. Public Health Nutr. 2023;26(8):1686–95. doi: 10.1017/S1368980023000320 36793234 PMC10410368

[39] de Haan S, Burgos G, Liria R. The nutritional contribution of potato varietal diversity in Andean food systems: a case study. Am J Potato Res. 2019;96(2):151–63.

[40] Borgi L, Rimm EB, Willett WC, Forman JP. Potato intake and incidence of hypertension: Results from three prospective US cohort studies. BMJ. 2016;:i2351. doi: 10.1136/bmj.i2351 PMC4870381 27189229

[41] Hu EA, Martínez-González MA, Salas-Salvadó J. Potato consumption does not increase blood pressure or incident hypertension in 2 cohorts of Spanish adults. J Nutr. 2017;147(12):2272–81. 10.3945/jn.117.252254 29046405

[42] Felix PV, Pereira JL, Fisberg RM. Associations between four diet quality indexes and high blood pressure among adults: Results from the 2015 health survey of Sao Paulo. Nutrients. 2024;16(5):629. 10.3390/nu16050629 PMC10935342 38474757

[43] Liu Z, Zhao D, Dai S. Inverse association between the global diet quality score and new-onset hypertension in adults: A nationwide cohort study. J Nutr. 2024;154(4). 10.1016/j.tjnut.2024.02.008 38360116

[44] de Koning L, Chiuve SE, Fung TT, Willett WC, Rimm EB, Hu FB. Diet-quality scores and the risk of type 2 diabetes in men. Diabetes Care. 2011;34(5):1150–6. doi: 10.2337/dc10-2352 21464460 PMC3114491

[45] Kauffman SAE, Averill MM, Delaney JAC, Lemaitre RN, Howard BV, Fretts AM. Associations of diet quality and blood serum lipoprotein levels in a population at high risk for diabetes: the Strong Heart Family Study. Eur J Clin Nutr. 2020;74(7):1084–90. doi: 10.1038/s41430-019-0539-1 31804627 PMC7272258

[46] Bromage S, Andersen CT, Tadesse AW. The Global Diet Quality Score is associated with higher nutrient adequacy, midupper arm circumference, venous Hemoglobin, and serum folate among urban and rural Ethiopian adults. J Nutr. 2021;151(Suppl 2):130S-142S. 10.1093/jn/nxab264 PMC8564694 34689198

[47] Huggins CE, Margerison C, Worsley A. Influence of dietary modifications on the blood pressure response to antihypertensive medication. Br J Nutr. 2011;105(2):248–55. 10.1017/S0007114510003223 20807467

[48] Shim JS, Jung SJ, Kim HC. Self-reported diet management, dietary quality, and blood Pressure control in Korean adults with hypertension. Clin Hypertens. 2023;25(1):24. 10.1186/s40885-019-0130-z PMC6911701 31890276

[49] Villarreal-Zegarra D, Carrillo-Larco RM, Bernabe-Ortiz A. Short-term trends in the prevalence, awareness, treatment, and control of arterial hypertension in Peru. J Hum Hypertens. 2021;35(5):462–71. 10.1038/s41371-020-0361-1 PMC8134053 32518303

[50] García-Molina L, Lewis-Mikhael AM, Riquelme-Gallego B. Improving type 2 diabetes mellitus glycaemic control through lifestyle modification implementing diet intervention: A systematic review and meta-analysis. Eur J Nutr. 2020;59(4):1313–28. 10.1007/s00394-019-02147-6 31781857

