# Supplementary material for: Diet quality and blood pressure among adults with hypertension living in a high-altitude region of the Peruvian Andes
Source: PLOS Glob Public Health. 2026 Oct 28;6(10):e0007396. doi: 10.1371/journal.pgph.0007396 (PMC13716217; doi:10.1371/journal.pgph.0007396)
Supplement: Supplementary file 1 [file pgph.0007396.s001.docx]

**SUPPORTING INFORMATION**

Table A. Daily energy intake by participants clinical characteristics in ANDES trial in 2024 (n=154)

|  | **Energy (kcal/day)**  **Median (IQR)** |
| --- | --- |
| Sex  Male  Female | 1,409 (1,019 – 1,952)  1,205 (753 – 1,531) |
| BMI (kg/m^2^)  Normal  Overweight  Obesity | 1,152 (740 – 1,776)  1,428 (993 – 1,849)  1,281 (742 – 1,568) |
| Hypertension awareness and treatment  No  Yes | 1,336 (834.9 – 1,747)  1,282 (854 – 1,605) |
| Hypertension control*  Uncontrolled  Controlled | 1,276 (867 – 1,973)  1,296 (841 – 1,560) |

*Hypertension control using the SBP/DBP < 140/90 mmHg threshold among those with prior hypertension diagnosis and treatment

Abbreviations: GDQS, Global Diet Quality Score; BMI, body mass index; SBP, systolic

Table B. Proportion of participants by GDQS levels of intake of all food groups in ANDES trial in 2024

| **GDQS food groups** | | **Proportion of participants (%)** | | | |
| --- | --- | --- | --- | --- | --- |
|  |  | **Low** | **Middle** | **High** | **Very high** |
| **Healthy** | Citrus fruits | 72.73 | 4.55 | 22.73 | 0 |
|  | Deep orange fruits | 99.35 | 0 | 0.65 | 0 |
|  | Other fruits | 61.04 | 9.09 | 29.87 | 0 |
|  | Dark green leafy vegetables | 87.66 | 8.44 | 3.9 | 0 |
|  | Cruciferous vegetables | 96.1 | 1.95 | 1.95 | 0 |
|  | Deep orange vegetables | 32.47 | 35.71 | 31.82 | 0 |
|  | Other vegetables | 27.92 | 42.86 | 29.22 | 0 |
|  | Legumes | 82.47 | 10.39 | 7.14 | 0 |
|  | Deep orange tubers | 98.05 | 0.65 | 1.3 | 0 |
|  | Nuts and seeds | 99.35 | 0 | 0.65 | 0 |
|  | Whole grains | 42.86 | 1.3 | 55.84 | 0 |
|  | Liquid oils | 64.29 | 15.58 | 20.13 | 0 |
|  | Fish and shellfish | 88.31 | 5.84 | 5.84 | 0 |
|  | Poultry and game meat | 46.1 | 11.04 | 42.86 | 0 |
|  | Low-fat dairy | 99.35 | 0.65 | 0 | 0 |
|  | Eggs | 69.48 | 9.74 | 20.78 | 0 |
| **Unhealthy if eaten excessively** | High-fat dairy | 73.38 | 16.23 | 9.74 | 0.65 |
|  | Red meat | 61.69 | 18.18 | 20.13 | 0 |
| **Unhealthy** | Processed meat | 99.35 | 0.65 | 0 | 0 |
|  | Refined grains and baked goods | 11.69 | 3.25 | 85.06 | 0 |
|  | Sweets and ice cream | 42.86 | 29.87 | 27.27 | 0 |
|  | Sugar-sweetened beverages | 83.12 | 5.19 | 11.69 | 0 |
|  | Juice | 84.42 | 0 | 15.58 | 0 |
|  | White roots and tubers | 18.18 | 25.97 | 55.84 | 0 |
|  | Purchased deep fried foods | 90.91 | 1.3 | 7.79 | 0 |

Abbreviations: GDQS, Global Diet Quality Score

Table C. Association between continuous GDQS and blood pressure by hypertension awareness and treatment in ANDES trial 2024

|  | **Blood pressure** | **p-trend** |
| --- | --- | --- |
| ***Not aware of and treated for hypertension (n= 48) **** | |  |
| SBP (mmHg)^‡^ | 0.24 (-1.5, 1.9) | 0.78 |
| DBP (mmHg) ^‡^ | 0.25 (-0.86, 1.38) | 0.65 |
| ***Aware of and treated for hypertension (n = 91) **** | |  |
| SBP (mmHg) ^‡^ | **-1.3 (-2.5, -0.1)** | **0.04** |
| DBP (mmHg) ^‡^ | -0.7 (-1.4, 0.1) | 0.11 |

*Adjusted models for age, sex, wealth score, food security status, years of education, occupation, BMI, physical activity, alcohol intake and glycemia HbA1c and “n” individuals with complete data on covariables

^‡^β-coefficient (95% Confidence Interval) of linear regressions

Abbreviations: GDQS, Global Diet Quality Score; SBP, systolic blood pressure; DBP, diastolic blood pressure
